# Fading beauty: The protein degradation mechanism behind rose petal senescence

**DOI:** 10.1093/plcell/koae069

**Published:** 2024-03-05

**Authors:** Peng Liu

**Affiliations:** Assistant Features Editor, The Plant Cell, American Society of Plant Biologists; Donald Danforth Plant Science Center, St. Louis, MO 63146, USA

Sending a bouquet of roses to a loved one is a profoundly romantic gesture. While the desire for these beautiful blooms to last forever remains unattainable, the process of their natural fading can be prolonged. In this issue, **Jingyun Lu, Guifang Zhang, and colleagues** (Lu et al. 2024) provide a comprehensive understanding of how rose petal senescence is controlled at the molecular level. In their study, a new rose protein, SENESCENCE-ASSOCIATED F-BOX (RhSAF), was identified as a key player in mediating ethylene-induced petal senescence by destabilizing proteins involved in gibberellic acid (GA) signaling.

Phytohormones, including ethylene and GA, intricately control various aspects of plant growth and development. Ethylene is recognized for its role in promoting petal senescence, whereas GAs function to repress this aging process (Ma et al. 2018). Ubiquitylation (also known as ubiquitination), a fundamental pathway for protein degradation in cells, involves ubiquitin ligases (or E3s), which direct the final transfer of the ubiquitin chain to the target proteins and are pivotal in most aspects of plant hormone signaling (Lechner et al. 2006; Kelley 2018). To search for ubiquitylation pathway genes that regulate flower senescence under ethylene influence, the researchers analyzed rose petal transcriptome data and found 19 F-box E3 protein genes upregulated under ethylene treatment. Among them, Rh*SAF* stood out because it displayed increased transcription and expression levels during flower opening and senescence. Additional assays confirmed that RhSAF was induced by ethylene treatment and suppressed by an ethylene perception inhibitor. Using virus-induced gene silencing (VIGS) to silence RhSAF in rose petals resulted in delayed petal senescence. Additionally, a stable transgenic RNA interference (RNAi) line exhibited a prolonged petal lifespan when Rh*SAF* expression was repressed. These findings suggest that RhSAF, induced by ethylene, plays a positive regulatory role in rose petal senescence.

The RhSAF protein has a conserved F-box domain at its N terminus, a nuclear localization signal, and a transmembrane domain at its C terminus. Utilizing a transient fluorescent labeling approach, the authors confirmed that RhSAF localizes to both the nucleus and plasma membrane. Interaction studies between RhSAF and component proteins of S-PHASE KINASE-ASSOCIATED PROTEIN 1 (SKP1)-CUL1-F-box (SCF) type E3 ligase complex identified *Rosa* SKP1-like 4 (RSK4), RSK7, RSK12, and RSK20 as interactors of RhSAF, strongly suggesting that RhSAF is indeed a component of the SCF complex in roses.

To look for target proteins ubiquitylated by RhSAF, the authors performed a yeast 2-hybrid screen using a petal cDNA library as prey. GIBBERELLIN INSENSITIVE DWARF1B (RhGID1B) and RhGID1C emerged among the identified proteins. The in-planta interaction between RhSAF and RhGID1B/C was further validated by several independent methods and paved the way for a more precise functional characterization of this interaction. Co-expression experiments of GID1B/C proteins co-expressed with either RhSAF or a truncated form of RhSAF lacking the key functional domain in *Nicotiana benthamiana* leaves strongly suggested that RhSAF promotes the degradation of RhGID1B/C proteins. GID1 proteins function as GA receptors; when bound to GA, they interact with the DELLA proteins that are master repressors of GA signaling (Willige et al. 2007; Shimada et al. 2008). Intriguingly, the findings of Lu et al. (2024) confirmed that RhSAF mediates the ethylene-induced degradation of RhGID1, leading to the accumulation of DELLA proteins and the suppression of GA signaling (Fig.).

**Figure.**
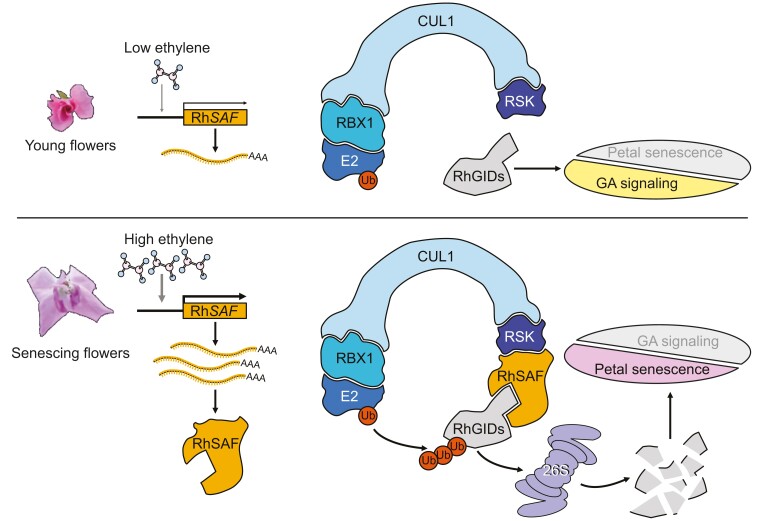
Model of RhSAF-regulated petal senescence in roses. In young flowers, low expression of *RhSAF* leads to the promotion of GA signaling. At a later stage of flowering, the increased level of ethylene induces *RhSAF* expression, which mediates the degradation of RhGIDs. The loss of RhGIDs represses GA signaling and accelerates petal senescence. Reprinted from Lu et al. (2024), Figure 8.

Next, the authors investigated the functions of Rh*GID1B/C* in petal senescence. Using VIGS to individually silence Rh*GID1B* or Rh*GID1C* had no discernible impact on flower senescence. However, when both genes were simultaneously silenced, petal senescence was accelerated. Notably, the accelerated flower senescence phenotype was complemented when RH*SAF* was also silenced alongside Rh*GID1B/C*. This indicates that RhSAF expedites petal senescence by destabilizing RhGID1B/C. Lu et al. further found that the protein levels of RHGID1B/C were decreased under ethylene treatment; however, this was a minor effect in the Rh*SAF*-RNAi plants, indicating that degradation of RhGID1B/C proteins promoted by ethylene is dependent on RhSAF.

Overall, the researchers established the essential role of the RhSAF-RhGID1 module in ethylene-induced petal senescence in roses. They elucidated the molecular-level mechanism by which ethylene antagonizes GA signaling in flower senescence, highlighting a ubiquitylation-dependent pathway. This study holds potential applications for extending the longevity of flowers in the ornamental flower industry.

## Dive Curated Terms

The following phenotypic, genotypic, and functional terms are of significance to the work described in this paper:

GID1b Gramene: AT3G63010
GID1b Araport: AT3G63010

GID1c Gramene: AT5G27320
GID1c Araport: AT5G27320
